# Multiculturalism in social agencies for the aged

**DOI:** 10.1186/2193-1801-3-429

**Published:** 2014-08-12

**Authors:** Thomas Akintayo, Juha Hämäläinen, Sari Rissanen

**Affiliations:** Department of Social Sciences, University of Eastern Finland, Kuopio Campus, P O Box 1627, Kuopio, FI-70211 Finland; Department of Social Sciences, University of Eastern Finland, Kuopio Campus, Kuopio Finland; Department of Health and Social Management, University of Eastern Finland, Kuopio Campus, Kuopio, Finland

**Keywords:** Aged care agencies, International social work, Multiculturalism

## Abstract

This study uses content analysis and visual representation methods to explore how multiculturalism is displayed on the websites of agencies providing social care for the aged. These agencies use strategically planned texts to portray multicultural categories of inclusion, diversity and individuality; and emphasize the text referents through ethno-related pictures as universal equivalence symbols for ethno-cultural diversity. With the few cases of non-text referents, which are open to cultural and sociological relativities, the study posited that the patterns and modes of portraying multiculturalism are similar. It concluded with the need for further studies to establish whether state policies or agencies’ market strategies are behind the liberal multicultural references.

## Background

While the debate on multiculturalism – a socially complex dynamism – is still raging, nevertheless multiculturalism is found in the everyday life of people across the globe. It has remained a powerful force in modern societies (Banting and Kymlicka, 2006), particularly in the cases of national minorities and indigenous people in traditional countries of immigration (Kymlicka, 2007; Scheffer, 2011) as evidenced in the United States, Canada, and Australia. Multiculturalism has neither been limited to scholarly philosophical debates, nor to policy issues in the political arena, but manifested in everyday social practices in both traditional and non-traditional countries of immigration all over the world.

One of such social practices is the social work profession, particularly international social work. In social work profession generally, Sue (2006) devoted a seemingly classic multicultural social work textbook to calling for organizational change in social work education and practices that would reflect not only traditional therapeutic practices, but also an emphatic focus on rebalancing clients in their familial and community contexts by means of treatment practices aimed at freeing them from cultural oppression. Similarly, Gutierrez (2001) reviewed scientific literature at developing an *Ethno Conscious Perspective* that focused on equity, equality and fairness and a social development agenda (a departure from the individualistic approach) in order to change the low status of ethnic minorities communities, refugees, and people of color in the United States. The reality of multiculturalism in the social work profession is asserted further by Longres (1997) who posited that multiculturalism is arguably the most important issue in providing for the wellbeing of people in ethno-culturally diverse settings. Thus multiculturalism works for *social justice* – a key principle in social work’s international definition (Adams *et al*., 2009). It encompasses social welfare for everyone. And in particular, Mayadas and Elliot (1997), p. 176 emphasized that 21^st^ century international social work has as its core values “globalization, transculturalism, multiculturalism, democracy, diversity, socio-cultural and ethnic exchange”. They apparently synthesized multiculturalism and international social work with the international communities and institutions; the globalization of the social work profession; identification with international institutions; and advocacy for local problems originating from the global system (Healy, 2001; Lyons *et al*., 2006; Cox and Pawar, 2006; and Hugman, 2010).

Within the seemingly wide scope of multiculturalism, the focus of this study is on agencies’ social care of the aged, in which the administration of agencies is contemporarily being affected by what Torres-Gil and Moga (2001), p. 14 described as the “growth of minority populations, continued immigration, increase in longevity, the aging of the baby boomer and public policy responses…”. Hence, the question arises of whether social care agencies for the aged have ever strategically reflected multiculturalism on their websites, either in the past, or contemporarily, as internet has increasingly become the gateway to social life, and as a response to increasing global social mobility. In a seemingly first attempt at providing answers to the question, due to the dearth of literature on this type of research, this study empirically explores how multiculturalism is being displayed on the websites of agencies providing social care for the aged. This is done towards a better understanding and advancement of international social practice of caring for the aged in ethno-cultural diverse settings, as well as in contexts resemblance of (hypothetical) *Banal Nationalism* “in which public institutions and public space are imprinted with a particular national identity” (Kymlicka, 2007, p. 64) thus depicting an illusion of ethno-cultural homogeneity that is immunized against social mobility.

Consequently, this study primarily uncovers the nature and pattern of how multiculturalism is portrayed by agencies providing social care for the aged, for replication and possible improvement. In addition, the study explores the commonality of a notion that meeting the needs specific to cultural identity (a strong factor in multiculturalism) is an indispensable factor for the wellbeing of the aged (Lai 2012), when organizing and strategizing social services for older people in ethno-cultural diverse settings. At achieving these goals, the background sub-sections illustrate further how the study has systematically used both the concepts of multiculturalism and that of agency for the aged, thereafter followed by literature review concerning the area of focus. The sections on results and discussion contain the empirical discoveries and their implications for international social work practices and social policies. The concluding section contains the limitation on the scope of the theory generated as well as recommendations for further studies, while the techniques deployed in the study are described in the section for methods.

### Conceptual framework

#### Multiculturalism

The concept of multiculturalism is apparently complex in meaning. Cumulative knowledge about human nature has shown that no two human beings are completely the same or different, and human societies are however made up of these differences. Their developments into a complex reality of socio-cultural environment is no longer under debate, as social research has revealed the world to be a composite of social evolution called multiculturalism, and the global interdependence of these socio-cultural composites is contemporarily being called ‘globalization’ (Dominelli, 2009). Nevertheless, Torres-Gil and Moga (2001), p. 16 asserted that the concept as used in social work “appears to reference the value and even desirability of encouraging racial and ethnic groups to maintain their group self-identities”. This is also the concept’s referent in major scholarly texts such as Banting and Kymlicka (2006) and Kymlicka (2007), but Sue (2006) added ‘feminism’ to the grouped referred to, and Jay (2002) also referred to ‘sexual orientation’. However, due to the varied meaning of multiculturalism there is the need to delve into the evolution of the concept so as to illustrate its usage in this study.

‘Multiculturalism’ shares conceptual complexity with its parent word – ‘culture’. Culture has been defined from the perspectives of three senses: as artistic or intellectual work by the humanities scholars; as a way of life by anthropologists and sociologists; and as a development process of historical documents and methods by historians (Baldwin *et al*., 1999). Similarly, the term ‘multi-culture’ became ‘multiculturalism’ for Dewing and Leman (2006), who argued that the concept has its origin, from Canadian government policy of 1965 and 1971. And also for Koleth (2010), pp 4 who asserted that it was used in Australia in 1973 “as the basis for migrant settlement, welfare and social-cultural policy”, hence multiculturalism has been seen from different perspectives: firstly as a *political prescription* (or ideology) that aims at legitimizing the incorporation of racial and ethno-cultural diversity in the general structure of any society; and secondly as a *social reality* that describes racial and ethno-cultural diversity as a phenomenon (Kallen, 1982). These perspectives fit into what Taylor *et al*. (1994) have viewed as ‘the *politics of recognition’* in his understanding of multiculturalism. In agreement with these lines of thought, Raihanah (2009) summarizes that multiculturalism has become a wide raging concept which can be used to describe an ideology, a social policy or aspects of public structure. Thus it can be inferred that within the discussion of ethno-racial relationships, particularly among the classical countries of immigration, the dominant argument is the political aim and agenda for integration.

In addition, Jay (2002) argued for the need to understand multiculturalism from different national historical perspectives, invariably it can also be inferred that the concept is a product of cultural diffusion that collocates with its variant models in state policies such as ‘social integration’ , ‘ethno-cultural diversity’ , ‘assimilation’ , ‘acculturation’ (Kallen, 1982; Torres-Gil and Moga, 2001; Kymlicka, 2007) and ‘cultural pluralism’, ‘melting-point’ , ‘race-relations’ , ‘identity politics’ (Jay, 2002), paving the way for, as much as embedded in, the theoretical arguments of cultural and sociological relativities. Therefore, from these perspectives this study defines *multiculturalism as the evolving multi-racial* and *ethno-cultural inclusion, diversity and individuality in everyday interactions that have effects on how social institutions are organized*. The definition is used in this study as the main theoretical framework giving focus and direction, and informs what to look for in the text contents of organizational strategies on the websites of sampled agencies.

#### Social care agencies for the aged

The word ‘aged’ generally refers to individuals in the final stage of their life-span (Johnson *et al*., 1997). Everyone, irrespective of the population group of belonging may contend with health-related problems, but the probability of developing health problems becomes more pronounced as one comes to the later stage of life (Heffernan *et al.*, 1988). Hence agency care for the aged is becoming complex in most societies, particularly in the advanced countries, owing to changes in family types, urbanization and high social mobility. Adding to these complexities is the changing racial and ethno-cultural demography of the elderly, which is one of the practice fields in which social workers play important helping roles globally.

Consequently, different models of service delivery and partnership are emerging as multipurpose senior centers in a seemingly new model of services for older people. This is an abridged form of the ‘social science models’ and the ‘bio-medical models’ (Payne, 2009) with emerging multicultural trends as portrayed in sample data, and are called here the ‘Multi-Racial & Ethno-Cultural Models’ of social care for the aged. They include *social services,* in which professional social workers assist the aged to make healthy and happy adjustments by solving social problems, thereby serving as advocates (particularly in residential care) and liaising between family members of clients and staff. That is, social workers *ethically* serve as client companions *all* the time, with a multi-racial and ethno-cultural approach. Other services are *therapeutic services*, which may be physical or recreational, (some in the domain of clinical social workers); *nursing services*; *medical services*, which include physicians, clinical specialists, dentists, podiatrists and ophthalmologists; and lastly *ancillary services* such as pharmacy, radiology, or clinical laboratory services.

Therefore, the concepts of agency social care for the aged are as simple as the social and medical models, and as complex as the emerging multi-racial and ethno-cultural models illustrated above, and together with the concept of multiculturalism as defined previously, serve as the conceptual and contextual frameworks for searching for relevant literature review, and for data gathering techniques in this study.

### Literature review

There is a vast ‘ethnogerontological’ literature reflective of care for the aged from multi-racial and ethno-cultural backgrounds (Brotman, 2003), particularly those focusing on care of the aged in the residual liberal welfare model contexts. Nevertheless, few of these studies are concerned with how ethno-cultural diversity is portrayed on the websites of agencies providing social care for the aged. For example, Holland and Katz (2010) highlights cultural provision in extra care for the elderly Jewish community in United Kingdom. Rose and Cheung (2012) asserted that literature review of 54 articles published between 2001 and 2011 across countries advocated the need to combine ethnic, cultural and ‘gerontological’ issues into the diagnostic and statistical manual of mental disorders for accurate assessment of each client. Dockery’s (2010) empirical study highlighted the welfare link between race, ethnicity and culture among indigenous Australians. In the United States Longres (1997) expressed a similar view that the major goal for minorities is to gain access to the resources that allow for success, particularly the welfare industry, as a means to an end. Thus the crucial role ethnic identity (and diversity) can play in the promotion of health and care of the elderly as asserted by Lai (2012) is expressed by all the studies but none of them is concerned with how multiculturalism is strategically entrenched and portrayed by the agencies.

In addition several non-empirical studies have been concerned with *culturally competent practice*. For example, in a systematic academic review of literature on delivery of elderly care in the culturally and linguistically diverse backgrounds of Australia, Radermacher *et al*. (2008) asserted in their findings that culturally competent practice is required to deliver a responsive and effective community aged care system, a view akin to views expressed in related studies in the United States (Dong, 2012; Browne and Mokuau, 2008; Gutierrez *et al.*, 1996), and in Sweden (Forssell and Torres, 2012). Their views confirmed Brotman’s (2003) earlier assertion though in the context of Canada that previous studies have been too focused on developing competency skills rather than on exposing and altering institutional structures and power relations dotted with racism.

Other studies have focused on a *programmatic dimension* to multiculturalism for social care of the aged in contemporary time. In California Chow *et al*. (2010) linked racial and ethnic variations to different services of support for adults of age 50 and above. Giunta *et al*. (2012) explored racial and ethnic diversity in senior centers in New York by comparing participants’ characteristics in more and less multicultural settings, and highlighted the importance of multicultural programming for senior centers as a result of demographic changes which seem bound to eliminate mono-cultural care centers for seniors. The focus of the study has also been directed towards *composite factors of multiculturalism* that are challenging care systems for the aged. Nguyen (2012) discovered English proficiency as the cause of access disparities to care services among Asian-American elderly immigrants to the United States. Bhattacharyya *et al*. (2012) also discovered that language and generational differences were some of the factors hindering effective service provision for the aged in the United Kingdom. The correlation between religion and ethnicity among African-American, Hispanic and White non-Hispanic was explored by Morano and King (2005), revealing the in-depth effects which multiculturalism factors can have on the care of the aged in culturally diverse settings. Also Alalauri and Hujala (2013) highlighted the growing importance of multiculturalism in the care profession by describing the discourses of multiculturalism on organizational culture and everyday life in care work community, including the elderly care contexts in Finland. However, agencies’ portrayal of multiculturalism on their websites is apparently out of focus in all these studies.

Nevertheless, few studies are conducted on multicultural dimension of care system structures but no information on how multiculturalism is portrayed by the agencies. In the United States, Torres-Gil and Moga (2001) compared senior center participants in an ethno-racially diversified setting with those in a non-diversified setting, and discovered that public policy responses could affect care system designs. This discovery is similar to Lum (2005) argument that most studies have neglected racial and ethnic differences in the structure and size of care giving networks in the United States. In the United Kingdom, the exploration of the mistreatment of black and minority ethnic groups by Bowes *et al*. (2012) uncovered structural and contextual factors to be more contributive than cultural factors for elderly care. Also, Health Service Executive (2005) structurally adopted a ‘Whole Organization Approach’ that embedded multicultural awareness, such as the usage of signage at the reception and public areas, to care for the languages of service users as a response to the challenges of ethno-cultural diversity in Ireland. Apparently none of these studies have considered their websites as a vital platform for disseminating multiculturalism strategically in view of its importance to social care delivery, even in culturally diverse settings.

Thus almost all the literature reviewed emphasized the importance of multiculturalism to the wellbeing of people in ethno-culturally diverse settings from theoretical, empirical, programmatic, clinical and cultural competence approaches. However, with the exception of studies by Torres-Gil and Moga (2001), Health Service Executive (2005) and Bowes *et al*. (2012), no other studies made reference to strategic and structural display of multiculturalism by agencies, particularly on their websites, in the care of seniors, who need a more sensitive approach to accessing social care, in an age where internet has increasingly become the gateway to accessing social services. Hence the current inquiry into how multiculturalism is portrayed on the websites of agencies providing social care for the aged.

## Results and discussion

### Results

In the course of transforming the data from qualitative to quantitative, the study uncovered that multimedia is used by many of the agencies to portray multiculturalism. In other words, a combination of ‘texts’ and ‘pictorial referents’ (– two variables together) are used by half of the cases to enhance the textual meanings of the multicultural issues they are concerned with. In some cases only the texts or photographic images are used as in the *‘Qualitative Analytic Matrix* of *Portraying Multiculturalism’* in Table 1 below:

**Table 1:** **Qualitative analytic matrix of portraying multiculturalism (n = 24)**

Categories of texts	***Agencies***& countries: their ***strategic statements***’ as text referents to multiculturalism	Pictorial referents
INCLUSION	*Highway Aged* in South Africa: *Mission* includes “We care for all older persons…”	Afro/Euro/Asia
	*Darlingford Upper Goulbourn Nursing Home* in Australia: *Values* include “For the individual and the right to self-determination for all.”	-
	*Ask Friendship* Centre in Canada: *Values* include “…all people have the right to…”	Euro/Asia
	*Community Care Durham* in Canada: “all people… be treated with… dignity and respect.”	Euro/Asia
	*Sunrise Senior Living* in US: *Mission* includes “…all seniors. …offering choices.”	-
	*Kateri Residence Skilled Nursing and Rehabilitation Center* in US: *Mission* includes “…enhances the comfort and dignity of all…”	Euro/Afro
	*Oxford Private Care* in UK: “Respect…choice are important to us…service for everyone.”	-
	*CHATS* in Canada: *Values* include “inclusivity” of everyone.	Euro/Afro/Asia
	*Loretto* in US: *Values* include “justice, inclusiveness…”	-
DIVERSITY	*Circle of Care* in Canada: *Values* include “diversity.”	Euro/Afro/Asia
	*Cobble Hill Health Center* in US: *Values* include “Embracing the cultural diversity of the communities we serve without regard to race, color, gender, religion, or national origin.”	Euro/Afro
	*Bergen Regional Medical Center* in US: *Values* include “We embrace cultural diversity…”	-
	*Lincoln Park Care Center* in US: “… not discriminate on the basis of race, color, religion, sex, national origin.”	Euro/Afro
	*Isabella* in US: *Mission* includes“…care… without regard to race, creed or nationality.”	Euro/Afro
	*Terence Cardinal Cooke Health Care Center* in US: *Mission* includes “…serve people of … races, creeds, economic means and ethnic backgrounds.”	-
	In US: “*Kings Harbor*'s resident community is diverse in background, culture, religion, race and age, and the care provided meets a broad range of needs …”	Euro/Afro
	*Philadelphia Nursing Home* in US: *Values* include “…maintains …& preserves a sense of freedom, identity, & independence.”	Euro/Afro/Asia
INDIVIDUALITY	*Friendship Ridge* in US: “…supports the individual needs of patients and families.”	Euro/Afro
	*Valley View Nursing Home* in US: *Philosophy* includes “Opportunities… for individuals to share values and to maintain their beliefs.”	-
NB: These agencies are with only pictorial referents to multiculturalism	*Aberdeen Health & Community Services* in Canada	Euro/Afro/Asia
	*Parker Jewish Institute* in US	Euro/Afro/Asia
	*Lifespan* in US	Euro/Afro
	*Savannah Grand* in US	Euro/Afro
	*Home Instead Senior Care in US (with Franchise in 13 countries for this study)	Euro/Afro/Asia

❖ Euro/Afro/Asia = refers to pictures of White Europeans, Black Africans and Asians respectively.

❖ - (dash) in the pictorial referent column means no pictures displayed by the agencies, but texts only.

❖ *Home Instead operates with franchise world-wide from the US, but with websites in English, in 13 countries.

Multiculturalism issues are entrenched in the agencies’ Strategic Planning Statements (SPSs) or texts, stating the reasons for the agencies existence and the social roles being performed in their respective countries of location. Organizational purposes, objectives, directions or long-range plans are referred to as SPSs (Bradford *et al*., 2000; Riggs, 1984), and in this study they are concerned with the statements of ‘*mission, vision, goal, value, philosophy, aims* and *others*’ , stipulating the essence of the agencies. And as portrayed in the Qualitative Analytic Matrix’s table, the SPSs exhibited similar multicultural sequences, patterns and frequencies that are categorized as multiculturalism of *inclusion, diversity* and *individuality*.

Firstly in the category of inclusion, the usage of the words: ‘all’ , ‘inclusivity’ and ‘everyone’ dominated all the texts, and featured in the following SPSs: mission, vision, value and others. This category is portrayed in nine cases located in South Africa, United Kingdom, United States, Canada and Australia, and five of the cases illustrated their textual referents with pictures of Africans, White Europeans, and people from Asia for emphases. Secondly, category of diversity is also filled with statements or texts referring to ‘cultural diversity’ , religion’ ‘independence’; and no discrimination on the basis of ‘nationality’ , ‘background’ color’ ‘race’ , ‘sex’ , and ‘age’ , as portrayed in six agencies – five in United States and one in Canada. These agencies also exhibited similar pictorial referents to Africans, White Europeans, and people from Asia. Lastly, the category of individuality featured ‘individuality’ in the value statements and statements of philosophy of two agencies located in the United States, with only one of the two exhibiting pictorial referents in addition to textual references as emphases of the multiculturalism issues within the agency.

As illustrated with the SPSs above, the pictures generally served as same equivalence of references for clarity and emphatic purposes across agencies and countries, but relative to the presence of particular ethnic groups in each agency’s location. Apart from being the concrete symbols of ethnic referents they also exhibited the same patterns across the categories of multiculturalism as used in this study. The pictures also served as the only medium of portraying multiculturalism in some cases, particularly as seen in one agency (Home Instead Senior Care) based in United States and operating on franchise to non-liberal welfare regimes.

### Beyond the texts and pictures focus

Findings beyond the use of multimedia or ‘texts’ and ‘pictures’ are uncovered as in the following: firstly, the quantity and the spread of agencies that strategically provided for the multicultural care of the aged across countries supported the position of Longres (1997) who generally asserted that the wellbeing of people in diverse settings are improved through multiculturalism, and the position of Mayadas and Elliot (1997) who argued that 21^st^ century would be years of globalization, and multiculturalism. Thus due to the expansion of globalization in the midst of increasing social mobility, however, an increase in multicultural activities should also be expected across the world.

Secondly, the twenty four (n = 24) samples are associated with the concept of liberal ideology. The agencies are all located in countries with liberal welfare regimes and residual models of social welfare policy (Esping-Anderson 1990). In addition, through franchise the possibility of portraying liberal multiculturalism in other welfare regimes manifested, even when there are no such textual references in those countries’ social policy documents. This is seen in the agency called “Home Instead Senior Care” that operates on *franchise* from United States to South Africa, Hong Kong, Quebec, Singapore, Chile, China/Belgium, France, Greece, Spain, Italy/Denmark, Sweden, and Norway respectively in the framework. Apart from South Africa and Quebec in Canada, these are countries operating different forms of social welfare regime, hence the argument that multiculturalism is of the liberal ideology.

In other words, the patterns of distribution exhibited in the use of the variables to portray multiculturalism at national and international levels are apparently linked to increasing ‘globalization of liberal multiculturalism’ (Kymlicka, 2007). The two variables – ‘text’ and ‘pictures’ – were assigned nominal numerical values to show their frequencies, and a more specific comparison of their dispersions in the data (Moore *et al*., 2012). This is illustrated in the quantitative content analysis in Table 2 which is the *‘Distribution of Text and Pictures in Aged Care Agencies’ Portrayal of Multiculturalism’* below:

**Table 2:** **Distribution of texts and pictures in aged care agencies’ portrayal of multiculturalism**

Multiculturalism categories	Variable frequency	
	Texts	Pictures
…of inclusion	9	5
…of diversity	8	6
…of individuality	2	1
Total=	19	12
THOSE THAT WERE NOT CATEGORIZED	-	
…due to lack of texts.	-	13
…lack of texts but with franchise	-	5
Total=	-	18

On one hand, the previous qualitative findings are confirmed by the quantitative table. Nineteen cases in the text column of the multicultural categories have twelve of the cases as textual referents, and they emphasized these referents with ‘pictures’ (multimedia). Thus the texts’ column represented the concrete social policies of the respective agencies, and each policy in the twelve cases is strongly emphasized by the supporting pictures. On the other hand, cases that used ethno-pictorial images ambiguously to portray multiculturalism are in the pictures’ column of the table. Among the eighteen uncategorized cases, where ‘pictures’ are the only referents to portray multiculturalism, there apparent ambiguity is uncovered as their references to liberal multiculturalism might have different meanings in non-liberal welfare regime countries. Hence those on franchise are seemingly used as extension of neo-liberalism to other types of welfare regimes, an apparent process of globalizing the liberal ideology, which would subsequently have multicultural implications. In other words, their usage can be subjected to ambiguous symbolic interpretations when there are no textual explanations of multiculturalism on their websites, or in their national social policy documents.

Therefore with this quantitative support for the preceding qualitative findings, this study empirically theorizes that *the patterns and modes of portraying multiculturalism by social care agencies for the aged are similar. These agencies use texts that consist of multicultural categories of inclusion, diversity and individuality in their strategic plans; and emphasize their text referents through ethno-related pictures as universal equivalence symbols for ethno-cultural diversity. The non-text referents to pictorial images, however, are open to cultural and sociological relativities. And these modes of portraying multiculturalism are apparently rooted and originated from the liberal ideology.*

### Discussion

This section focuses on the commonality of the notion of multiculturalism across countries and continents; the implications of the study for social work practices; the minimal nature of the theory posited; and the need for further studies. Apart from discovering how social care agencies for the aged are portraying multiculturalism on their websites, the scope of the sampling technique used in this study helps to uncover multiculturalism as *a global wide-spread notion vital to the wellbeing of the aged* particularly among those in ethno-cultural diverse societies. Examples of studies expressing this view ranges from those conducted in the traditional countries of immigration and to others in non-traditional countries of immigration. Those in traditional countries of immigration include that of Dockery (2010) in Australia, Lai (2012) in Canada, and Torres-Gil and Moga (2001) in United States – these are the countries where majority of the samples used in this study are located. Those conducted in non-traditional countries of immigration include that of Holland and Katz (2010) in United Kingdom, Health Service Executive (2005) in Ireland, Alalauri and Hujala (2013) in Finland, and Forssell and Torres (2012) in Sweden. In addition, the notion was also expressed in a study conducted at a global level by Rose and Cheung (2012), hence the notion is a global phenomenon and the modes of portraying multiculturalism as demonstrated by the cases can be adapted globally.

The practice implication of the modes for portraying multiculturalism, first and foremost, is the use of multimedia (a combination of texts and pictures) as methods of communication by the agencies. It solves conceptual ambiguity in bi- or multi- lingual settings at ensuring welfare access and availability to all ethnic groups in both existing and emerging multicultural societies, and which can be adopted at national and global social policy levels. For example, the communication models justify the context in which the social care agencies from Canada are operating as it is a reflection of the demographic characteristics of the country given the assertion of Masi and Disman (1994) that prior to 1950, about 90% of immigrants to Canada came from Europe, and thereafter the percentage has decreased to less than 20%, and similarly argued by Lai (2012) that the percentage of people of color in Canada have increased from 4.7% in 1981 to 16.2% in 2006. Thus new Canadians now come from such diverse areas as East Africa, South East Asia, Central America, South America, and South Asia”.

Secondly, the modes are also of importance to social work practice, in the contexts of global interdependence of multi-ethnic populations, as an institutional multicultural structure of communication that has universally understood symbols of referents. Such communication models will contribute to the wellbeing of clients psychologically, in terms of confidence at accessing services and assurance of their availability for everyone. Therefore by using the communication models social work profession can further reject the double-jeopardy hypothesis attributed to cultural diversity (Lai 2012) which has been argued to be detrimental to the wellbeing of the aged in ethno-culturally diverse contexts.

## Conclusion

In conclusion, though the study covers major visible ethno-cultural populations, such as the non-Caucasian in race; the colored people; and the Aborigines, yet the use of the findings is however limited on the ground that this study is seemingly the first to investigate how multiculturalism is portrayed on the websites of agencies providing social care for the aged. In addition, the use of non-probability sampling and the apparent limitation imposed by using English as the only language for selecting the samples in the midst of other international language contexts can also be considered as limiting the purview of the theory generated by the study.

Nevertheless, the mapping of words and phrases within the full domain of the multiculturalism framework and the availability of sampled agencies used in the study for replication provide the *content validity* necessary (Neuendorf, 2002), as research validity represents the truthfulness of the data. Additionally, if subsequently replicated through the same processes and procedures, it would support the *reliability* of the study. Thus the quest of this study to understand how multiculturalism is portrayed on the websites of agencies revealed the complexities inherent in the concept as embedded in the national and international politics of diversity. It is also discovered that the aged care agencies make reference to multiculturalism irrespective of whether their national contexts have adopted multiculturalism as state policies. Hence these raise the question of whether these agencies’ displays of multiculturalism on their websites are mandated by state policies or organization market strategies. Whichever way further studies are needed, as organizational cultures are sometimes claimed to be different from national cultures, and multiculturalism as well has been asserted to have been adopted as a national policy in some countries.

## Methods

The Convenience Sampling (Rubin and Babbie, 2008) of the non-probability sampling method was used for this study, due to the difficulty of obtaining a global directory of organizations providing social care for the aged to enhance probability sampling technique. Therefore, the search-words: “Organizations Providing Elderly Care” with the addition of ‘Africa’, ‘Asia’, ‘Australia’, ‘Europe’, ‘North America’, ‘South America’ and name of countries on separate occasions via search engine – *Google.com.* – were used, and they conveniently generated sixty five (n = 65) agencies in English across countries. However, after initial observation of the samples, twenty four (n = 24 across countries) made references to multiculturalism, and they are all located in countries of liberal welfare regime and residual social policy model. In addition one of the twenty four samples is based in the United States operating on franchise in other countries of varied welfare regimes.

At analyzing the twenty four samples further this study used a mixed-method of content analysis and visual method to investigate how multiculturalism is portrayed on the websites of those agencies. Firstly, qualitative content analysis examined the ‘texts’ on the websites for patterns, similarities, differences and categories (Miles and Huberman, 1994; Rubin and Babbie, 2008). Secondly, the photographs on the websites were also subjected to their visual representations of multiculturalism (Banks, 2001; Denscombe 2003). Thus both the ‘texts’ and the ‘photographs’ were analyzed simultaneously (Miles and Huberman, 1994) to examine if their referents are directed to same objectives. Lastly, quantitative content analysis (Neuendorf, 2002) was used to describe the distribution of the variables to support and make additions to the findings of the qualitative content analysis.

Focusing on the ‘texts’, the study used the concept of ‘multiculturalism’ in the global diffusion of societies, as previously defined in this study to encode and categorize statements referring to ‘inclusion’, ‘diversity’ and ‘individuality’ at identifying multiculturalism issues. Also by focusing on the pictures on the same websites the visual images used as pictorial referents to people of color, the Aborigines, and the Caucasians are identified and coded as – ‘Afro’ for Black Africans; ‘Euro’ for White Europeans; and ‘Asia’ for people from Asia, that is, the Chinese and the Indians recognized by their facial features and in some cases by their dressing styles.

## Authors’ information

This article is just one, out of others, to be produced towards the award of a PhD in social work. The study program by Thomas Akintayo is targeted at developing a new theory and/or model of multiculturalism in international social work. And the program is under the supervision of Professor Juha Hämäläinen and Professor Sari Rissanen.
